# Gender differences in work-related high mobility differentiated by partnership and parenthood status

**DOI:** 10.1007/s11116-021-10226-z

**Published:** 2021-09-25

**Authors:** Isabelle Wachter, Christian Holz-Rau

**Affiliations:** Department of Spatial Planning, Transport Research Group, TU Dortmund University, 44227 Dortmund, Germany

**Keywords:** Long-distance commuting, Overnighting, Gender, Parenthood, Interaction terms

## Abstract

The income situation and the division of labor in households, which are closely related to occupational mobility, are central aspects of the debate on gender equality. Women have shorter commuting times and distances than men and spend fewer nights away from their main place of residence for work-related reasons. Various studies attribute these gender differences to a gendered division of labor and the associated greater involvement of women in household tasks and childcare. Consequently, studies investigating these gender differences focus primarily on employees in relationships and the associated intra-couple interactions, while little attention is paid to singles. Based on the German Family Panel (pairfam) this research aims to broaden the scope of interpretation and examines gender differences in work-related high mobility among employees in partnerships with and without children and among singles. Logistic regression models including gender interaction terms show that gender differences exist not only among employees with partners (and children), but also among singles. The results highlight that gender differences in high mobility are due to factors related to relationships and parenthood, as well as from other factors. Gender differences in high mobility are thus not merely the result of negotiation processes or of (patriarchal) power structures in relationships and gendered labor division. They are also related to gendered occupational segregation and economic disparities and internalized gender preferences that are independent of partnership and parenthood.

## Introduction

The income situation and the division of labor in households are key aspects of the debate on gender equality. Both are closely linked to work-related mobility, which connects the professional and domestic spheres. Previous research has consistently shown that women have shorter commutes than men in terms of distance (Crane 2007; Sandow and Westin 2010; Axisa et al. 2012) and time (European Communities 2004; McQuaid and Chen 2012). Furthermore, women spend fewer nights away from their main residence due to work (Collet and Dauber 2010; Reuschke 2010a; Rüger et al. 2011). Traditional gender roles, where men are the wage earners and women are responsible for household tasks and childcare, are considered one of the main drivers of this gender difference. Accordingly, the majority of research focuses on couples (with children) and associated intra-household decisions (Plaut 2006; van der Klis and Mulder 2008; Gimenez-Nadal and Molina 2016). Only a few studies address these gender differences among singles (households). They mostly indicate smaller gender differences among singles (Mauch and Taylor 1997; Chidambaram and Scheiner 2020; Hu 2020).

Based on that, this research examines gender differences in work-related high mobility (daily long-distance commuters and overnighters) among persons in relationships with and without children and among singles based on the German Family Panel (pairfam). Additionally, we investigate how individual and household-related attributes interact with gender in terms of high mobility. This approach aims to identify how factors related to partnership and parenthood, as well as other factors, are linked with work-related high mobility and thus expand the scope of interpretation of gender differences in high mobility.

In the next section, we summarize the theoretical perspectives and previous research on gender differences in work-related mobility. This is followed by the “Data and methodology” section and the “Results” section. We conclude with  the “Discussion and conclusions” section, where we also point out limitations and further research needs.

## Theoretical background and literature review

### High mobility: long-distance commuting and overnighting

Definitions of *daily long-distance commuting* (LDC)^1^ vary depending on study area and focus. Distance-based definitions have a considerable range, e.g. 17 km per way in Canada (Maoh and Tang 2012), 30 km in Sweden (Sandow and Westin 2010) and 50 km in Germany (Statistisches Bundesamt and Wissenschaftszentrum Berlin für Sozialforschung 2018). Definitions based on travel time often set the threshold at 60 min one-way or at an overall commute time of 120 min^2^ (Schneider et al. 2001, Schneider and Meil 2008, Vincent-Geslin and Ravalet 2016). While there are many studies on LDC and commuting times and distances in general, there is less research on *overnighters*. The study Job Mobilities and Family Lives in Europe (JobMob) (Schneider and Meil 2008, Schneider and Collet 2010) and further work partly based on the JobMob data (Rüger et al. 2011; Ravalet et al. 2014; Viry and Kaufmann 2015) examine several forms of job-related spatial mobility including LDC and overnighting.

Overnighters often spend nights away from home due to work-related reasons, with the threshold usually set at 60 nights per year (Huynen et al. 2008; Rüger et al. 2011; Ravalet et al. 2015). Overnighting subsumes a variety of mobility forms and thus includes *shuttles*, who are also known as weekly or monthly long-distance commuters, and *vari-mobiles,* who travel frequently and at irregular intervals to changing work locations. Schneider and Meil (2008) and Viry and Kaufmann (2015) also classify people in long-distance relationships as overnighters. In contrast to couples living apart together (LAT), their reasons for not sharing a common household with their partners are professional rather than private.

Although LDC, shuttles, and vari-mobiles differ in the aspects mentioned, they are similar in important aspects for the present study. This includes that all three forms of mobility constitute a strategy to reconcile the own personal, social and professional life and—if applicable—additionally that of the partner and children (Limmer and Schneider 2008; Andreotti et al. 2013). All three types of mobility are very time-intensive and therefore have a high impact on other life domains as they reduce the available time for them (Limmer and Schneider 2008; Lück and Ruppenthal 2010; Ravalet et al. 2015). Thus, they “are the result of space and time trade-offs between personal and professional lives” (Ravalet et al. 2015). Moreover, there are gender differences in each of the three types of high-mobility. These gender differences, which are the focus of our study, are explained by the same theories that we are going to discuss in the following section. For these reasons, like Viry and Kaufmann (2015), we use the term *high mobility* to summarize LDC, shuttles and vari-mobiles.

### High mobility and gender differences

Despite tendencies of convergence, analyses show that women commute shorter distances and times than men (Crane 2007; Konrad 2016). Furthermore, they are more rarely overnighters (Green et al. 1999; Collet and Dauber 2010; Reuschke 2010a; Rüger et al. 2011). Explanations for these gender differences exist in various scientific disciplines. In the following, we present the most common hypotheses and explanations.

*Gender roles* and the resulting gender-specific division of labor are considered one of the main reasons for gender differences in travel and commuting behavior. The greater involvement of women in household tasks, including childcare, limits women in time and space, which manifests itself in shorter commutes and less frequent overnighting compared to men. This is also known as the Household Responsibility Hypothesis (HRH) (Hanson and Johnston 1985; Johnston-Anumonwo 1992; Turner and Niemeier 1997; Silveira Neto et al. 2015; Gimenez-Nadal and Molina 2016). Despite rising female employment rates, social gender roles remain largely unchanged (Coltrane 2000; Warren 2003). Accordingly, women undertake more household-support and child-serving trips than men (Schwanen 2007; Liu et al. 2012; Fan 2017). In line with the HRH, Chidambaram and Scheiner (2020) find that gender differences in commuting in dual-earner households are lower if the male partner spends more time on unpaid work. Nevertheless, the majority of studies examine gender differences among heterosexual couples. Smart et al. (2017) find that gender differences also exist in same sex couples with respect to labor division and travel, but they are less pronounced.

Other explanations focus on *occupational segregation* and *economic disparities*. Female-dominated occupations are more evenly distributed in space than male-dominated jobs, which favors short commutes (Hanson and Johnston 1985; Singell and Lillydahl 1986; Hanson and Pratt 1988). Moreover, various studies have found a positive association between education and income and high mobility practices (Schwanen et al. 2004; Lück and Ruppenthal 2010; Sandow 2014). Full-time employees also commute longer than part-time employees (Zolnik 2010; Axisa et al. 2012; Maoh and Tang 2012) and are disproportionately represented among overnighters (Green et al. 1999; Bonnet and Collet 2010; Reuschke 2010b). However, women generally have lower incomes and are more likely to work part-time, with the differences in Germany being especially large in an EU comparison (European Institute for Gender Equality 2014; Boll and Lagemann 2018), which can cause gender differences in high mobility. Accordingly, Madden's results (1981) indicate that gender balance in terms of working hours and wages would lead to equal or even longer commutes for women. Other studies show that although gender differences decrease when income and occupation are controlled for, they still remain (Gordon et al. 1989; Rüger and Sulak 2017).

Income is also an important parameter in *family and household economics* (Mincer 1978; Becker 1991). Mincer (1978) considers migration (e.g. residential relocation) decisions based on the total (in)material benefits and costs of all household members. If total migration benefits exceed total migration costs, the household relocates. If the costs outweigh the benefits, e.g. if one partner’s income gain from a migration cannot (over)compensate the loss of income of the other partner, the household does not relocate and high mobility becomes more relevant. Thus, the decision-making process in households regarding relocation and high mobility is more complex in multi-person households. High mobility is sometimes the only way to combine two careers and a common place of residence (in the preferred residential area) (Green 1997; Vincent-Geslin and Ravalet 2016). Nisic (2010) points out that individual efforts (commuting/(un)paid work) and benefits related to household decisions are unevenly distributed among household members. Thus, a household’s consensus is the result of *negotiation processes*, whereby the relative position of power in partnerships determines which interests prevail (Blau 1986). Empirical studies often measure this position of power using the ratio of incomes (Nisic 2010). As mentioned above, women in general have lower incomes and thus weaker bargaining positions than their partners.

Feminist theories trace gender differences back to *patriarchal power relations*, which shape gender roles and relationships. Patriarchal power relations hold women responsible for household tasks, thus restricting their freedom and promoting gender inequality (Fenstermaker and West 2002). This gender hierarchy privileges men (Wood and Eagly 2002) and serves as an orientation framework for (household-internal) decisions. Thus, such power relations exceed the economic differences described above (Hartmann 1976; Walby 1994).

This is in stark contrast to other theories, which Fan (2017) summarizes as theories of *internalized gender differences*. These theories explain gender differences in gender-specific personalities, attitudes and preferences (Hakim 2000). Empirical evidence indicates for example that women have greater environmental concerns and responsibility (Davidson and Freudenburg 1996) and are more inclined to decrease their car use than men (Matthies et al. 2002; Polk 2004), which could explain the lower work-related mobility of women. However, these gender differences in attitudes and preferences could also result from gender-specific socialization. Moreover, Reuschke and Houston (2020) find, after controlling for household responsibility, gender differences in commuting time among employees, but not among self-employed persons who are less affected by labor market constraints. They therefore conclude that labor market constraints play a more important role than gender preferences.

Most of the hypotheses and explanations for gender differences in high mobility presented indicate some disadvantages/constraints for women. According to the hypothesis of patriarchal power relations, prescribed gender roles, and economic inequalities and the often related lower bargaining power in the household negotiation process, women are primarily responsible for domestic labor, although this may not correspond to their preferences. This leads to temporary and to spatial constraints for women, which in turn limits their mobility, activity spaces, access to labor markets, and economic independence. However, there are also numerous burdens and disadvantages associated with high mobility. Rüger and Ruppenthal (2010) and Sandow et al. (2014) discuss the disadvantages of LDC and overnighting in detail, we therefore only briefly address the disadvantages of high mobility practices. High mobility results in increased levels of stress (Novaco et al. 1991; Gottholmseder et al. 2009). Moreover, Sandow et al. (2014) find a higher mortality risk for women who have experienced LDC than for women with shorter commuting distances, but they do not find this association for men. Additionally, high mobility practices shorten the time available for other activities, resulting in unhealthy habits such as less sleep and exercise or unhealthy eating habits, which over time can cause health issues (Christian 2012). Longer commuting times are also associated with a higher risk of work-life conflicts (Hämmig et al. 2009), especially for women (Jansen et al. 2003). According to the JobMob study, overnighters in particular perceive a lack of time for their partner and children as a disadvantage of their high mobility (Rüger and Ruppenthal 2010). Due to the disadvantages mentioned above and the negative environmental impact of high mobility, we question whether it is desirable for women to be as highly mobile as men. For this reason, we avoid the normative term “gender gap” and prefer “gender differences”.

### Gender differences in high mobility in the context of relationship status and household

Most of the explanations and hypotheses reviewed in the previous section explain the cause of gender differences in high mobility in terms of division of labor, which results from gender roles, negotiation processes and/or in patriarchal power relations. It is therefore likely that gender differences in high mobility are related to partnership and parenthood.

Female long distance commuters, shuttles and vari-mobiles are less likely to be in a partnership or to be married than both their male counterparts and women without high mobility practices (Rüger et al. 2011). Accordingly, married women have shorter commutes than men and other women without children (Preston et al. 1993; Lee and McDonald 2003; Preston and McLafferty 2016). In contrast, there is no clear association between relationship status and high mobility for men (Rüger et al. 2011). Some studies even find an increase in commuting distance and time with marriage for men (Preston and McLafferty 2016). Consistently, gender difference in commuting tends to be smaller among one-adult households without children than in other household types (Mauch and Taylor 1997; Hu 2020). Preston and McLafferty (2016) do not identify any gender differences in commuting time among unmarried persons without children. Zolnik (2010) finds no correlation between relationship status and commute time. Fan (2017) also concludes that parenthood rather than the pure presence of a partner is associated with larger gender differences in commuting times.

Women who practice long-distance commuting or overnighting are less likely to have children compared to other women and men (Schneider and Limmer 2008; Meil 2010; Reuschke 2010b; Rüger et al. 2011; Rüger and Sulak 2017), and mothers commute less than other women (Preston et al. 1993; Sandow 2008) as well as men in general (Schwanen et al. 2004). For men, on the other hand, the negative correlation between parenthood and high mobility is lower (Sandow 2008, Rüger and Sulak 2017) or does not exist at all (Rüger et al. 2011). Hu (2020) differentiates between two-adult households with one and two workers and finds that having children generally does not change gender differences in one-worker households but increases gender differences in two-worker households. However, Johnston-Anumonwo (1992) argues that the number of workers in a household rather than the presence of children is related to gender differences in commuting, and finds that the commuting distances of men and women differ more in dual-worker households than in single-worker households. In contrast, more recent studies (Sultana 2005; Fan 2017) find no association between breadwinner status and gender differences in commute time.

Regarding the age of children, Sandow (2008) finds a negative association between children under six and commuting distance for both women and men, while children over six are negatively associated with commuting distance only for women. Hjorthol (2000) also identifies that women with preschool-age children in particular work closer to home. Reuschke and Houston (2020) find the opposite with respect to commute time. In their study, women with the youngest child under five years commute significantly longer than women without dependent children. However, among parents, the age of the children has no significant effect on commute time. Gimenez-Nadal and Molina (2016) also find that age of children does not appear to be a relevant factor in commute times for men with children, while mothers with children under 5 years and 5 to 12 years tend to commute slightly longer.

As noted above, numerous studies confirm gender differences in high mobility. However, multivariate analyses usually include only the main effects of explanatory variables. Separate regressions for men and women are often used to identify gender-specific associations between independent variables and high mobility or commuting in general (Sandow 2008, Rüger and Sulak 2017). Interactions with gender are rarely part of multivariate analyses, although they test whether gender moderates the effect of independent variables on high mobility. The few analyses that consider these interactions are usually limited to certain variables (e.g. Reuschke 2010a; Rüger et al. 2011; Hu 2020). Moreover, most studies focus on high mobility in couple households (e.g. Plaut 2006; van der Klis and Mulder 2008). In general, they do not contrast their results with differences in high mobility between male and female singles, though possible differences among singles would indicate gender differences that go beyond dynamics in relationships.

Therefore, the subsequent analysis examines the following hypothesis: high mobility differs between women and men not only in relationships with and without children but also among singles, although they do not have to coordinate with or adapt to a partner. Thus, our analysis broadens the scope of interpretation of gender differences in high mobility among people in relationships, since the differences between men and women then have to be interpreted as the sum of influences dependent on and independent of relationships and parenthood. At the same time, this means that a deeper explanatory context of female and male decision-making processes has to be used to expand the hypotheses of gender roles, family economics, negotiation processes and patriarchal power relations. Additionally, we investigate how individual and household-related attributes interact with gender and how these interactions differ between singles and people in partnerships with and without children.

## Data and methodology

### Data

This research uses the German Family Panel pairfam (“Panel Analysis of Intimate Relationship and Family Dynamics”) Release 10.0 (Brüderl et al. 2019). This multidisciplinary annual long-term study started in 2008 and focuses on partnership and family dynamics among persons of the 1971–73, 1981–83 and 1991–93 birth cohorts. A special feature of pairfam is the multi-actor approach: in addition to the randomly selected persons—the ‘anchors’—their partners are also interviewed. This provides an extraordinary amount of information about the anchor and, where applicable, about the partner (see Huinink et al. 2011 for a detailed description of the study). As the present research examines gender differences in work-related mobility, it only uses survey waves 1, 3, 5 and 7 to 10, which contain information on commuting time. Due to the research focus, the present study only refers to anchors with the primary activity status “full-time-employed” or “part-time-employed”. Given the fact that self-employed people have greater control over their choice of workplace (van Ommeren and van der Straaten 2008; Reuschke and Houston 2020), generally have shorter commutes, and gender differences in commuting time are significantly smaller or not significant (e. g. Chidambaram and Scheiner 2020; Reuschke and Houston 2020), we decided to exclude them from the analyses. Unfortunately, the pairfam study does not include all the information listed in Table 1 to identify the partners’ mobility behavior.^3^ For this reason, the anchors’ partners are not included in the analyses as separate cases. Thus, the analyses are limited to employed singles and to employed anchors in partnerships regardless of the partner’s labor force status. Consequently, the study examines gender differences in high mobility among anchors across different households rather than gender differences within partnerships.

Since the number of highly mobile employees in the single survey waves is too small for a longitudinal analysis, we conducted a cross-sectional analysis, pooling data from the seven survey waves including commuting time. Accordingly, the data may include up to seven observations of the same anchor person. The pooled sample includes a total of 51,470 observations of anchors. 26,211 of these observations relate to anchors without the primary activity status “full-time-employed” or “part-time-employed” and are therefore excluded. Based on the research approach explained in the following section, 943 observations of employed singles with children are also excluded from the analyses as well as 124 observations from anchors with missing information about relationship status and children in the household. The final sample therefore encompasses 24,192 (weighted 22,201) observations.

### Methodology

As "High mobility and gender differences" and "Gender differences in high mobility in the context of relationship status and household" outline, the most widespread hypotheses and existing research evidence identify a strong link between partnership and parenthood and gender differences in high mobility. This suggests that gender differences in high mobility arise mainly (although not exclusively) from transformations in the partnership and parenthood context. Ideally, a longitudinal approach would be required to investigate the complex process of becoming highly mobile, which involves decisions across several temporal scales and involves different domains of life, e.g. residential and job location. While our data is not suited to conduct a longitudinal study ("Data"), we portray three important life stages with regard to gender differences in high mobility by dividing the employees into three groups differentiated by partnership and parenthood status. The first group consists of employees with partners and children (living in the same household) and the second group of employees with partners but without children. These two groups also include anchors who do not live with their partners in the same household, as we assume that these partners also influence the anchors’ mobility.^4^ The third group consists of single employees without children. For each of these groups, we conduct cross-sectional analyses and compare the results between the groups. This enables us to draw conclusions about gender differences in high mobility that are (in)dependent of partnership and parenthood. While gender roles, patriarchal power relations, family and household economics and the associated negotiation processes are expected to be particularly important for employees with partners and children, singles without children can decide mostly independently and do not have to balance their own interests against those of their partners and children (Hu 2020). Thus, gender differences among singles are more likely to be associated with internalized gender differences, occupational segregation and economic disparities, which may also be shaped by patriarchal structures, than with the other theoretical approaches. They thus serve as a kind of benchmark. Unfortunately, due to small sample sizes, we have to refrain from the separate analysis of one- and two-worker couples and we have to exclude singles with children from the analysis.

Binary logistic models for each group estimate the probability of high mobility. However, the non-independent (clustered) observations resulting from the pooling violate a fundamental requirement of “ordinary” regression models. Ignoring this dependency would result in errors in inferential statistics (Hu et al. 1998). Thus, we use the Generalized Estimating Equation Methodology (GEE), which takes the interdependencies of observations from one person into account using a work correlation matrix of within-subject dependencies as part of the model. The presented analyses are performed with a robust variance estimation algorithm and the working correlation matrix type "independent" (see Zorn 2001; Ghisletta and Spini 2004; Agresti 2007 for a detailed description of the GEE methodology). The interpretation of the GEE model presented here corresponds to that of ordinary logistic regressions. However, there is no coefficient of determination for GEE models. SPSS provides the Quasi Likelihood under Independence Model Criterion (QIC), which is an extension of the Akaike Information Criterion (AIC) for repeated measurements. The Corrected Quasi Likelihood under Independence Model Criterion (QICC) penalizes small sample size and model complexity. Models with smaller QIC(C) are better. (Garson 2013) We provide the QIC(C) for our models and the intercept models. In any case, there is no formal test of significance for model improvement. Therefore, similar to Scheiner and Holz-Rau (2012,2017), we present the R^2^ values of ordinary logistic regressions for comparison.

The study focuses on gender difference in high mobility and thus also on how individual and partnership-related attributes interact with gender concerning high mobility. Therefore, we also consider two-way interaction terms of gender with each independent variable. Formal interaction analysis using product terms in a single equation is preferable to separate regressions for men and women, as interaction terms formally test the difference between the logistic coefficients (Jaccard 2009). However, due to the high level of complexity, we refrain from a three-way interaction of gender, each independent variable and the three groups of employees. Instead, regression models are calculated separately for each of the three groups of employees and the regression models are contrasted. The inclusion of the interaction terms in part leads to high multicollinearity, but according to Jaccard (2009) this is uncritical. Furthermore, the gender interactions subdivide the samples by gender and, with the classification of the employees in the three groups mentioned above, lead to some small samples, which affects the p-values. This is especially true for strongly gendered attributes such as division of childcare or income constellation. Due to the explorative character of the study, we therefore focus more on the coefficients than on the significances in the models with interaction terms.

### Variables

“Highly mobile” (yes/no) is the dependent binary variable (Table 1). The operationalization of highly mobile persons is based on the literature ("High mobility: long-distance commuting and overnighting") and data availability and defines LDC based on commute time, as it can be assumed that time is more crucial than distance in the context of the compatibility of work, partnership and possible parenthood. Missing information about trip chaining on the commute is a limitation, as it is uncertain whether the commute time includes household-support or child-serving trips.^5^ Furthermore, commute distances are not included in the data and due to lack of data, we cannot distinguish LAT from long distance relationships, which is why LATs are not considered as overnighters as in Rüger et al. (2011). The other employed persons, who are neither long-distance commuters nor overnighters, serve as the reference group.

**Table 1:**
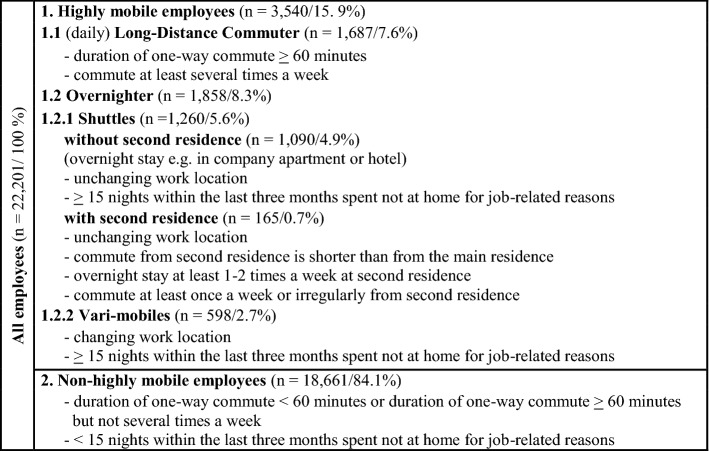
Operationalization of the dependent variable “Highly mobile”

Besides gender, the independent variables reported in Table 2 are included in the analysis. The process of deciding for or against high mobility is extremely complex and involves many domains of life. Focusing on gender differences in high mobility, we primarily include those variables considered most relevant for gender differences by existing research and hypotheses. Based on the assumption that the association between high mobility and other factors is largely the same for men and women, we do not include further variables. The division of labor within couples, especially the division of childcare, plays an important role for gender differences in high mobility ("High mobility: long-distance commuting and overnighting"). Assuming that one’s own perceived "workload" of childcare has a stronger influence on mobility behavior, we based the variable on the anchor’s perception.^6^ We prefer the income constellation division to differentiating between one- and two-income partnerships due to the importance of bargaining power in negotiation processes ("High mobility and gender differences"). Although desirable, subdividing into the four income constellations sole, main, equal and secondary wage earner possible due to the small number of observations, especially for male employees who earn less than their partners. For reasons of comparability, the reference categories of the two variables correspond as closely as possible to the situation of singles and childless anchors in partnerships. Other relative measures (e.g. educational differences between partners) considered in Motte-Baumvol et al. (2017) or Chidambaram and Scheiner (2020) are not part of the analysis as we aim to compare employees in partnerships and singles.

**Table 2:** Overview of utilized variables and their definitions

		Categories	Description/initial categories	% (Column)	Expected correlations with high mobility
*Dependent variable: high mobility (n = 22,201)*		Yes	Long-distance commuter or overnighter	15.9	
		No (ref.)	Others	84.1	
Variable for grouping: (n = 22,201)		Employees with partner and child	Married/with partner with a least one child living with anchor (cohabitating and non-cohabitating)	43.7	
		Employees with partner w/o child	Married/with partner without child living with anchor (cohabitating and non-cohabitating)	35.2	−
		Singles w/o child	Singles without child living with anchor	21.2	−
*Independent variables*					
Sex (n = 22,197)		Male		54.9	
		Female (ref.)		45.1	−
At least one child < 6 years (n = 9,697)		No (ref.)	All children living with anchor are older than 5 years	70.8	
		Yes	At least one child living with anchor is 0–5 years old	29.2	/^b^
Division of labour	Division of childcare (n = 9,138)	Mainly partner (ref.)	(Almost) completely my partner/for the most part my partner/another person	39.1	
		Egalitarian	Split about 50/50	34.4	+
		Mainly anchor	(Almost) completely me/for the most part me	26.5	+
	Income constellation (n = 21,350)	Sole or main wage earner (ref.)	Singles/anchors with a partner who is unemployed, in education or a housewife/houseman/all anchors who contribute ≥ 60% of the household income	55.9	
		Equal or secondary wage earner	All anchors who contribute < 60% of the household income	44.1	/^b^
Socio-economic status	Personal monthly net income (n = 20,959)	< €1,200 (ref.)		22.3	
		€1,200–1,699		26.6	+
		€1,700–2,299		25.7	+
		> €2,300		25.5	+
	Highest educational level (n = 22,186)	< University (ref.)	No degree, vocational training, vocational school, technical school, civil service training, technical college	80.2	
		> University	University, doctoral degree	19.8	+
Occupation	Temporary employment contract (n = 21,768)	No (ref.)		86.4	
		Yes		13.6	+
	Employment sector^a^ (n = 21,760)	White-collar (ref.)	Isco 08 major groups 1 (managers, skill level 3 + 4), 2 (professionals, skill level 4) and 3 (technicians and associate professionals, skill level 3)	50.6	
		Blue and green-collar	Isco 08 major groups 0 (armed forces occupations, skill level 1 + 2 + 4), 6 (skilled agricultural, forestry and fishery workers 2), 7 (craft and related trades workers, skill level 2), 8 (plant and machine operators and assemblers, skill level 2) and 9 (elementary occupations, skill level 1)	23.9	−
		Pink-collar	Isco 08 major groups 4 (clerical support workers, skill level 2) and 5 (services and sales workers, skill level 2)	25.5	−

^a^Based on Lee et al. (2016), whereby the main group “armed forces occupations” is assigned to the male-dominated blue and green-collar sector

^b^The findings of previous studies do not indicate a definite association between this variable and high mobility (see "Gender differences in high mobility in the context of relationship status and household")

## Results

### Descriptive analysis

The descriptive results, subdivided into the three groups of employees and gender (Table 3), are largely consistent with the literature. In general, men are more often highly mobile than women. Singles are less frequently highly mobile (males 17.8%, females 11.8%) than childless employees in partnerships (males 20.5%, females 15.2%), which applies slightly more to women. While the proportion of highly mobile men remains approximately the same with parenthood (21.1%), it is lowest among women with partners and children (6.7%). Accordingly, gender differences in high mobility are greatest among employees in partnerships with children and are less pronounced among childless employees in partnerships and singles, whereby the gender differences between the groups without children differ only slightly. This generally also applies within the categories of the independent variables.

**Table 3:** Descriptive summary across employees differentiated by gender, partnership and parenthood

	With partner and child				With partner w/o child				Singles w/o child			
	Male		Female		Male		Female		Male		Female	
	Total	Share of highly mobiles	Total	Share of highly mobiles	Total	Share of highly mobiles	Total	Share of highly mobiles	Total	Share of highly mobiles	Total	Share of highly mobiles
Total	5,286	21.1%	4,411	6.7%	3,957	20.5%	3,847	15.2%	2,949	17.8%	1,747	11.8%
*At least one child < 6 years*												
No (ref.)	3,382	22.5%	3,480	8.3%	-	-	-	-	-	-	-	-
Yes	1,904	18.6%	931	6.3%	-	-	-	-	-	-	-	-
*Division of childcare*												
Mainly partner (ref.)	3,455	22.7%	118	15.0%	-	-	-	-	-	-	-	-
Egalitarian	1,613	18.6%	1,527	8.9%	-	-	-	-	-	-	-	-
Mainly anchor	81	10.9%	2,344	4.8%	-	-	-	-	-	-	-	-
*Income constellation*												
Sole or main wage earner (ref.)	3,828	22.2%	518	9.3%	1,889	21.3%	1,004	13.5%	2,949	17.8%	1,747	11.8%
Equal or secondary wage earner	1,097	16.1%	3,649	6.3%	1,960	20.2%	2,706	16.1%	-	-	-	-
*Personal monthly net income*												
< €1,200 (ref.)	240	12.6%	2,254	3.6%	418	15.9%	791	8.1%	584	12.5%	376	8.3%
€1,200–1,699	743	11.9%	882	8.5%	1,100	14.5%	1,229	11.7%	968	15.8%	643	11.3%
€1,700–2,299	1,347	19.2%	549	12.4%	1,226	21.0%	1,077	20.5%	765	22.3%	418	13.5%
> €2,300	2,648	25.6%	451	10.8%	995	29.8%	579	22.9%	449	21.4%	220	18.6%
*Highest vocational degree*												
< University (ref.)	3,438	19.7%	3,098	6.2%	2,759	18.3%	2,534	13.8%	2,354	16.8%	1,206	10.2%
> University	1,845	23.9%	1,312	7.8%	1,194	25.7%	1,312	17.9%	592	21.7%	538	15.5%
*Temporary employment contract*												
No (ref.)	4,845	21.2%	3,768	6.9%	3,297	20.4%	3,173	14.7%	2,338	17.2%	1,377	9.8%
Yes	329	24.6%	552	5.9%	584	22.2%	626	16.1%	544	21.6%	331	18.9%
*Employment sector*												
White-collar (ref.)	2,565	24.7%	2,453	8.5%	1,827	24.5%	2,302	16.2%	950	24.4%	922	11.8%
Blue and green-collar	1,855	18.6%	426	2.8%	1,292	18.4%	228	6.9%	1,223	15.4%	173	8.1%
Pink-collar	773	15.0%	1,457	4.5%	750	15.1%	1,237	14.5%	699	11.9%	625	12.8%

Employees with at least one preschool-age child are less likely to be highly mobile (males 18.6%, females 6.3%) than employees with older children (males 22.5%, females 8.3%). Employees with greater involvement in childcare are less highly mobile, especially women. Women with main childcare responsibilities are considerably less likely to be highly mobile (4.8%) than women who share childcare with their partners in an equal way (8.9%) and women whose partners are mainly responsible for childcare (15.0%). Thus, gender differences—measured by the ratio of highly mobile men to women—become larger with higher involvement in childcare. Regardless of gender, employees with partners and children who are sole or main wage earners are more often highly mobile (males 22.2%, females 6.3%) than equal or secondary wage earners (males 16.1%, females 6.1%). By contrast, there is a heterogeneous pattern among childless employees in partnerships. While there is no clear relation between income constellation and high mobility for men, female equal or second wage earners without children are more often highly mobile (16.1%) than female single or main wage earners without children (13.5%).

The proportion of highly mobile people tends to increase with income, especially among women, so gender differences are generally smaller among employees with higher incomes. In addition, employees with high vocational degrees are more often highly mobile. The same is true for employees with temporary contracts except for women with partners and children (5.9% with vs. 6.9% without temporary contract). However, the proportion of temporary employees with partners and children is very low compared to the other groups. Especially for female singles, temporary employment seems to be associated with greater probabilities of high mobility (18.9% vs. 9.8% without temporary contract). Male employees in the white-collar sector are most likely to practice high mobility, followed by employees in the blue and green-collar and pink-collar sectors. The situation for women is more incoherent: among women with partners and children, employees in the white-collar sector are most frequently highly mobile (24.7%). In contrast, female employees without children in the white- and pink-collar sector are almost equally highly mobile. Consequently, the proportion of highly mobiles among childless male and female employees working in the pink-collar sector is nearly balanced. In the following section, regression analysis aims to disentangle these correlations.

### Multivariate analysis

Table 4 displays ordinal regression models for the three groups of employees differentiated by partnership and parenthood. As the literature indicates one-tailed hypotheses for most of the independent variables (Table 2), Table 4 contains mostly one-tailed p-values. Division of childcare and age of child(ren) are only relevant for employees with a partner and children and are included in Model 1. For the sake of comparability with childless employees, Model 2 does not consider these variables. Models 1 and 2 for employees with a partner and children reveal largely the expected correlation with similar magnitude with one exception: consideration of the division of childcare in Model 1 noticeably reduces the Exp(B) of gender.^7^ However, even taking childcare into account, men are significantly more often highly mobile than women, all else being equal (Exp(B) > 1). Thus, while the share of childcare is negatively related to high mobility, it only contributes to a limited extent to gender differences.

**Table 4:** Logistic regression analyses for high mobility differentiated by partnership and parenthood

	With partner and child								With partner w/o child				Singles w/o child				With partner and child								With partner w/o child				Singles w/o child				
	Model 1				Model 2				Model 3				Model 4				Model 1a				Model 2a				Model 3a				Model 4a				
	Exp (B)		Sig.^a^		Exp (B)		Sig.^a^		Exp (B)		Sig.^a^		Exp (B)		Sig.^a^		Exp (B)		Sig.^a^		Exp (B)		Sig.^a^		Exp (B)		Sig.^a^		Exp (B)		Sig.^a^		
Intercept	0.087		0.000		0.063		0.000		0.094		0.000		0.091		0.000		0.080		0.000		0.057		0.000		0.061		0.000		0.044		0.000		
Male *(ref. Female)*	1.692		0.003		2.120		0.000		1.412		0.003		1.644		0.007		2.724		0.039		3.178		0.004		3.613		0.000		5.193		0.000		
*At least one child* < *6 years (ref. No)*																																	
Yes	0.802		0.060^b^		-		-		-		-		-		-		1.129		0.562^b^		-		-		-		-		-		-		
Male * yes	-		-		-		-		-		-		-		-		0.656		0.089^b^		-		-		-		-		-		-		
(Men: yes)	-		-		-		-		-		-		-		-		(0.740)		-		-		-		-		-		-		-		
*Division of childcare (ref. Mainly the partner)*																																	
Egalitarian	0.929		0.279		-		-		-		-		-		-		0.611		0.117		-		-		-		-		-		-		
Mainly anchor	0.590		0.010		-		-		-		-		-		-		0.378		0.012		-		-		-		-		-		-		
Male * egalitarian	-		-		-		-		-		-		-		-		1.562		0.303^b^		-		-		-								
Male * mainly anchor	-		-		-		-		-		-		-		-		1.494		0.529^b^		-		-		-		-		-		-		
(Men: egalitarian)	-		-		-		-		-		-		-		-		(0.955)				-		-		-		-		-		-		
(Men: mainly anchor)	-		-		-		-		-		-		-		-		(0.565)				-		-		-		-		-		-		
*Income constellations (ref. sole or main wage earner)*																																	
Equal or secondary	0.856		0.225^b^		0.852		0.185^b^		1.146		0.195^b^		-		-		1.442		0.205^b^		0.962		0.867^b^		1.329		0.089^b^		-		-		
Male * equal or secondary	-		-		-		-		-		-		-		-		0.530		0.051^b^		0.852		0.562^b^		0.794		0.289^b^		-		-		
(Men: equal or secondary)	-		-		-		-		-		-		-		-		(0.764)				(0.820)				(1.055)				-		-		
*Personal monthly net income (ref.* < *1,200 €)*																																	
€1,200–1,699	1.721		0.003		1.753		0.002		1.245		0.074		1.386		0.041		2.483		0.000		2.477		0.000		1.620		0.015		1.669		0.054		
€1,700–2,299	2.480		0.000		2.650		0.000		2.225		0.000		1.950		0.002		3.708		0.000		3.891		0.000		3.444		0.000		2.398		0.020		
> €2,300	3.127		0.000		3.367		0.000		3.134		0.000		2.197		0.004		2.856		0.003		2.968		0.001		4.038		0.000		3.838		0.004		
Male * €1,200–1,699	-		-		-		-		-		-		-		-		0.376		0.004		0.369		0.003		0.517		0.015		0.789		0.272		
Male * €1,700–2,299	-		-		-		-		-		-		-		-		0.404		0.011		0.403		0.011		0.376		0.002		0.742		0.277		
Male * > €2,300	-		-		-		-		-		-		-		-		0.732		0.253		0.725		0.238		0.514		0.044		0.437		0.088		
(Men: €1,200–1,699)	-		-		-		-		-		-		-		-		(0.933)				(0.914)				(0.838)				(1.317)				
(Men: €1,700–2,299)	-		-		-		-		-		-		-		-		(1.499)				(1.568)				(1.293)				(1.780)				
(Men: > €2,300)	-		-		-		-		-		-		-		-		(2.091)				(2.153)				(2.076)				(1.676)				
*Highest vocational degree (ref.* < *university)*																																	
> University	0.775		0.067		0.723		0.022^c^		0.883		0.229		0.933		0.401		0.497		0.025^c^		0.481		0.015^c^		0.796		0.181		1.289		0.274		
Male * ≥ university	-		-		-		-		-		-		-		-		1.756		0.165		1.799		0.066		1.193		0.302		0.534		0.117		
(Men: ≥ university)	-		-		-		-		-		-		-		-		(0.873)				(0.865)				(0.949)				(0.688)				
*Temporary contract (ref. No)*																																	
Yes	1.319		0.056		1.292		0.064		1.423		0.003		1.770		0.000		0.712		0.114		0.802		0.189		1.481		0.015		2.570		0.003		
Male * yes	-		-		-		-		-		-		-		-		2.509		0.009^b^		2.110		0.023^b^		0.982		0.944^b^		0.586		0.170^b^		
(Men: yes)	-		-		-		-		-		-		-		-		(1.786)				(1.693)				(1.455)				(1.505)				
*Employment sector (ref. White-collar)*																																	
Blue and green-collar	0.754		0.038		0.719		0.023		0.854		0.159		0.749		0.095		0.363		0.003		0.361		0.002		0.509		0.040		1.171		0.373		
Pink-collar	0.581		0.001		0.560		0.001		0.896		0.243		0.715		0.086		0.571		0.024		0.553		0.013		1.133		0.282		1.616		0.111		
Male * blue and green-collar	-		-		-		-		-		-		-		-		2.406		0.039^b^		2.460		0.019^b^		1.672		0.225^b^		0.472		0.162^b^		
Male * pink-collar	-		-		-		-		-		-		-		-		1.023		0.949^b^		1.100		0.785^b^		0.553		0.057^b^		0.244		0.003^b^		
(Men: blue and green-collar)	-		-		-		-		-		-		-		-		(0.849)				(0.888)				(0.851)				(0.553)				
(Men: pink-collar)	-		-		-		-		-		-		-		-		(0.584)				(0.608)				(0.627)				(0.394)				
QIC, intercept	8,085				8,085				7,342				4,066				8,085				8,085				7,342				4,066				
QICC, intercept	8,053				8,053				7,337				4,060				8,053				8,053				7,337				4,060				
QIC	6,109				6,413				6,439				3,627				6,095				6,399				6,437				3,620				
QICC	6,068				6,379				6,401				3,585				6,035				6,350				6,375				3,555				
R^2^ (Cox & Snell)^d^	6.9				6.6				3.0				2.5				7.6				7.0				3.6				3.4				
R^2^ (Nagelkerke)^d^	12.2				11.7				4.9				4.3				13.4				12.6				5.9				5.9				
n (observations)	10,061				10,789				6,601				4,073				10,601				10,789				6,601				4,073				
n (individuals)	3,426				3,598				3,140				2,000				3,426				3,598				3,140				2,000				

^a^one-tailed p-values ^b^two-tailed p-values ^c^not significant due to the one-tailed *p*-values/hypothesis of a positive association between a high vocational degree and high mobility ^d^R^2^ of ordinary logistic regression

In line with the results of Reuschke and Houston (2020), Model 1 shows no significant association between the age of children and high mobility among parents. As assumed, higher income and employment in the white-collar sector is positively associated with high mobility (Model 1 and 2). In contrast, the models do not confirm the expected positive correlation between high mobility and a high vocational degree while taking income constellation, income and the employment sector into account.^8^ Furthermore, employees with a partner and children on temporary contracts are not significantly more likely to be highly mobile, which could be due to the low proportion on temporary contracts ("Descriptive analysis"). Additionally, the income constellations in partnerships do not have any significant correlation with high mobility for employees with partners and children.

Model 3 for childless workers in partnerships and Model 4 for singles show similar but weaker correlations regarding gender and income. The same is true for employment sector, especially the pink-collar sector, but the associations are not significant for the two groups without children. In contrast to Models 1 and 2 for employees with children, the positive association between high mobility and temporary contracts is significant for childless employees. Similarly to employees with partners and children, there is no significant correlation between a high vocational degree and the income constellation with high mobility in the other groups.

In general, the models that include only main effects confirm most of the expected correlations. Regardless of group classification, the known gender differences in high mobility are significant, although they are highest among employees with partners and children (Model 2). However, when controlling for childcare (Model 1), the gender differences in this group are similar to those observed among childless employees. Moreover, it is striking that single women who have little or no need for consultation or coordination with a partner are also less likely to be highly mobile than men (Model 4). These gender differences even seem to be slightly greater than for childless workers with partners (Model 3).

The interaction terms in Models 1a–4a provide further findings for interpretation. Due to the gender interaction, the conditional main effects indicate the Exp(B) only for women and the respective gender interaction terms the Exp(B) for men relative to women. Accordingly, their product equals the Exp(B) for men, which are shown in brackets in Table 4. Furthermore, due to the interactions with gender, the main conditional effect of gender refers only to persons with the characteristics of all reference categories and thus to a very specific group of persons. Consequently, the conditional main effect of gender is only of limited relevance. Due to the large number of possible combinations of characteristics considered here, we refrain from presenting the gender differences for each of them and concentrate on whether the gender interaction terms with the respective characteristic increase (Exp(B) > 1.0) or decrease (Exp(B) < 1.0) gender differences in high mobility. As discussed in "Methodology", we focus primarily on the effects and less on the significances.

To exemplify the interpretation, consider Model 3a for childless employees with partners. The odds of high mobility for female employees in the highest income category (> €2,300) are 4.038 times higher than the odds for female employees in the lowest income category (reference category < €1,200). The Exp(B) of the interaction term male* > €2,300 is 0.514 and considerably weakens the conditional main effect for men. Consequently, the Exp(B) for male employees in the highest income category are 4.038*0.514 = 2.076. Thus, especially for women, a high income has a positive correlation with high mobility, which also affects gender differences: for employees with characteristics of all reference categories, including an income below €1,200, the conditional Exp(B) of gender is 3.613. Keeping all else unchanged except the income, the Exp(B) of gender for employees in the highest income group is 3.613*0.514 = 1.857 and hence substantially lower.

Models 1a and 2a for employees with partners and children including gender interaction terms again show similar correlations. The majority of the conditional main effects are stronger for women than in the main-effect Models 1 and 2 (Exp(B) deviate further from 1) and are weakened for men by gender interactions (Exp(B) are closer to 1). The conditional effect of gender decreases when childcare is taken into account. However, as mentioned above, this effect is linked to many characteristics and is therefore of limited informative value.

Model 1a indicates a slightly positive tough not significant association between the presence of children younger than 6 years old and high mobility for women. For men, this association seems to be rather negative. Furthermore, Model 1a shows that even if women and men contribute the same amount of childcare, childcare is to a greater extent negatively associated with women’s high mobility and thus increases gender differences. Egalitarian childcare seems to be negatively related to high mobility only for women. If the anchor is mainly responsible for childcare, this is associated with lower probabilities of high mobility, especially for women. By controlling childcare in Model 1a, gender differences tend to be smaller for equal and secondary wage earners, as the status of equal and secondary wage earners tends to be positively associated with high mobility for women, while the opposite seems to be the case for men. Models 1a and 2a indicate that high income is associated with high mobility, particularly for women. Thus, gender differences in higher income groups are smaller than in the lowest. The models also indicate that the positive association of temporary contracts with high mobility in the main-effect Models 1 and 2 is due to the association of men. Only men with a temporary contract seem to be more likely to be highly mobile, but not women. On the other hand, the negative correlation between employment in the blue and green-collar sector and high mobility that was identified in the main-effect Models 1 and 2 is primarily present among women.

The respective gender interactions vary between the groups of employees subdivided by partnership and parenthood status, whereby all interaction terms for singles are associated with lower gender differences (Exp(B) < 1). The comparison of the models including gender interactions reveals different patterns. The first pattern is visible for personal net income. Regardless of group, income is more positively associated with high mobility for women, whereby gender interaction tends to decline with childlessness and single status (with the exception of the highest income class). The declining interaction effects are mainly due to the declining positive correlation of income and high mobility among women, while the Exp(B) of income among men differ less across the groups of employees. This pattern also occurs for the income constellation when childcare is considered. Here, too, the interaction of gender and status as equal or second earner is slightly more pronounced among employees with children (Model 1b) than among employees without children (Model 3b) and is thus more strongly correlated with a decrease in gender differences among the former.

A reverse pattern occurs in the highest income category. Here the interaction with gender increases with childlessness and single status. However, the number of female employees within the highest income class is limited (Table 3). The third pattern concerns the highest level of vocational qualifications, temporary contracts and employment sector. While the respective gender interaction terms are positive (Exp(B) > 1) for employees with partners and children, who are associated with higher gender differences compared to the respective reference groups, the intensity of the positive interactions effects decreases and becomes the opposite with childlessness and single status (Exp(B) < 1). Once again, this is primarily due to the changed association of female employees in the groups of employees. Female singles with temporary contracts are significantly more mobile than female singles with permanent contracts. For men the correlation seems to be much weaker. Moreover, the lower probability of high mobility among employed singles in the green and blue as well as in the pink-collar sector compared to white-collar sector, which was found in the main-effect Model 4, is thus limited to single men. Accordingly, gender differences in high mobility among single employees with a high vocational degree, temporary contract and employment in the blue and green-collar and in the pink-collar sector are lower than in the respective reference categories.

Measured by R2 of conventional regressions, the model fit is highest for the models of the employees with a partner and children. At 12% they are low, but typical for studies of transport behavior at the individual level (Chidambaram and Scheiner 2020). For childless employees with and without partners, the R2 value is approximately as low. For all groups, the inclusion of interaction terms slightly increases the model fit.

## Discussion and conclusions

This research examines gender differences in high mobility. To broaden the scope of interpretation of gender differences in high mobility in couples, we also include single employees in the analyses. Furthermore, using gender interaction terms as part of regression models allows us to infer how division of labor and socioeconomic and occupational characteristics interact with gender in terms of high mobility, considering different life stages. Thus, our research combined with existing and future research can provide the basis for more precise target-group specific policy implications.

When interpreting the results, attention should be paid to the explanations and hypotheses for gender differences in high mobility, but the assessment of high mobility ("High mobility and gender differences") also has to be considered. High mobility practices can be a strategy for a career start or professional advancement in certain professions and could facilitate access to (more attractive) jobs. In this sense, high mobility is an advantage and the lower rate of high mobility among women may indicate restrictions in their professional careers. However, whether high mobility is an advantage also depends on the type of career. Future research with a more differentiated subdivision of occupations than occupational sectors could provide further insights. Nevertheless, high mobility is also associated with social and health burdens. A possible alternative to high mobility is relocation, which may in turn lead to a greater need for high mobility of the partner or to uprooting the family. Therefore, the decision for highly mobile practices instead of relocation relieves not only the partner but also the children, who can stay in the desired or familiar residential and working environment. In this sense, high mobility is a way of combining career and partnership or family, but it is also a burden for those who practice it. The lower level of high mobility among women should therefore not be interpreted exclusively as a disadvantage.

The results presented do not provide definitive evidence on how gender differences in high mobility should be evaluated. However, they partially support the perception of high mobility as a burden. According to the descriptive results, singles who are largely independent in their decisions are less likely to be highly mobile than childless employees with partners. This applies to both men and women, indicating that they avoid high mobility if possible. A relationship generates stronger spatial ties, which employees may compensate for with high mobility. Therefore, and according to the descriptive results, both genders are more likely to be highly mobile in partnerships without children than singles, although this is slightly more the case for women. Consequently, gender differences are marginally smaller among childless employees with partners than among singles. The multivariate analyses also point to these smaller gender differences. Children in the household increase the local attachment even more. The descriptive as well as the regression models indicate that the proportion of employees with highly mobile practices remains about the same among men, whereas the proportion for women is considerably lower. As a result, gender differences are greatest among employees with partners and children. Taking childcare into account reduces these gender differences and thus supports HRH and the demand for policy action to support the reconciliation of work and family life. To facilitate this reconciliation and avoid high mobility, policy makers and companies could offer in addition to comprehensive childcare services, more flexible working conditions for employees. These include working from home, flexible working hours and the (partial) replacement of face-to-face meetings and the associated business trips by video conferences. As the COVID-19 crisis has shown, this is possible to a high degree in many, though not all, occupations.

However, it is worth noting that the direction of causality is not clear. On the one hand, workers might avoid high mobility to care for children; on the other hand, high engagement in childcare might counteract high mobility. Future studies could investigate causality using structural equation modeling. Nevertheless, significant gender differences remain when childcare is considered, which could be attributed to patriarchal power relations and the gendered division of labor besides childcare in couples. However, as these remaining gender differences have approximately the same magnitude as for singles, it is reasonable to assume that these gender differences are due to gender-specific factors that are already at work for singles. These differences may be based on occupational segregation and economic disparities, or to a general lower acceptance of women towards high mobile practices (internalized gender preferences). If the latter were the case, women would reject high mobility more than men and the lower level of high mobility of women in partnerships would constitute an advantage for women.

The regression models including gender interaction terms provide supplementary findings for interpretation. Although the results are rather exploratory in nature ("Methodology") and the described differences between the employment groups differentiated by partnership and parenthood still need to be tested for significance (e.g. by using three-way-interactions, "Variables"), the results point to gender-specific differences that intensify or diminish with increasing bonding through a partnership and parenthood. These changes arise primarily from alterations in the associations for women, suggesting that partnership and parenthood are particularly associated with female travel behavior.

The gender interaction with temporary contracts is interesting for the evaluation of high mobility as a (dis)advantage. Among singles, temporary contracts have a much higher positive correlation with high mobility for women than for men. Conversely, this means that women in permanent employment avoid high mobility more than men and mainly practice high mobility under the conditions of temporary contracts. For female employees with partners, the correlation between temporary contracts and high mobility is much less pronounced and at about the same level as for men. It seems that temporary contracts are a less decisive factor for high mobility than the compatibility of a relationship and (two) professional career(s). By contrast, among employees with partners and children, a positive correlation between temporary contracts and high mobility exists only for men. This could be due to and consistent with the gender role of men as “bread-winners” in that men (usually the main earners) who have temporary contracts and are in partnerships with children prefer to practice high mobility rather than to uproot the family from its familiar environment. However, the proportion of temporary employees among employees with children is considerably lower, implying that employees do not choose to have children until they have “more secure” (permanent) employment arrangements. Considering high mobility as a burden and as a strategy to bridge uncertain situations, both the policy makers and the companies could aim to reduce temporary employment where possible.

Gender-specific economic disparities could be one reason for the stronger positive correlation of income with high mobility for women, even among singles. Thus, a better-paid job offers an incentive for high mobility, especially for women. In this sense, reducing or, ideally, overcoming gender economic disparities could reduce incentives for high mobility for women and the associated burdens. The partnership-independent differences intensify with increasing attachment to partners and children primarily due to the increasing associations of women’s income with high mobility. This might point to the importance of women’s income within the family and household decisions and in the associated negotiation processes. The gender interaction with the income constellation also tends to increase with children (when childcare is controlled for) and implies that female equal and secondary wage earners are more often highly mobile than their male counterparts. It is likely that the household income or the income of the couple is higher than that of sole or main wage earners. In this sense, the results are consistent with Chidambaram and Scheiner (2020), who found that higher household income is associated with longer commuting times for women and thus with lower gender differences. They attribute this on the one hand to more pronounced equal intra-couple economic power relations, and on the other hand to a possible self-selection effect, as mothers who work might be more career-oriented, which in turn is related to longer commutes. Here, a differentiation between equal and second earners would certainly be informative, but is not possible due to the small sample size ("Variables"). However, the fact that the status of equal or secondary earners is associated with more high mobility for women could also be due to maintenance and child-supporting trips on the way to work. These trips are more frequent for women than for men and cannot be distinguished from "pure" commutes to work based on pairfam ("Variables"). This, in addition to the self-selection effect, might also explain why the presence of preschool children tends to be positively related to high mobility for women, but not for men.

The finding that working in the pink-collar sector tends to be positively associated with high mobility for female singles but not for male singles weakens the hypothesis of occupational segregation. However, the gender interaction with the pink-collar sector changes with increasing attachment to partners and children. Accordingly, there is no noteworthy interaction between gender and the pink-collar sector for employees with partners and children. Again, primarily for women, the effects vary differentiated by partnership and parenthood. This indicates that it is not occupational segregation per se that is linked to gender differences in high mobility, but rather the compatibility of employment in the pink-collar sector and high mobility with partnership and parenthood. For example, specific working conditions such as late or changing working hours can counteract this compatibility. The policy, but also the companies themselves, should thus promote the compatibility of family and career, especially in the pink-collar sector. However, relative characteristics in partnerships may also explain why working in the pink-collar sector is associated with a lower likelihood of high mobility for mothers. A more detailed examination of these relative measures in couples that are decisive for family and household decisions could provide further insights. We have refrained from this in favor of comparability with singles, but pairfam offers further possibilities to investigate this in more detail.

The results, differentiated by gender as well as by partnership and parenthood, indicate that gender differences in high mobility are related to partnership and parenthood as well as to other factors. Gender differences in high mobility are thus not only a result of negotiation processes, of a (patriarchal) power structure in relationships or of gendered labor division. They are also an expression of internalized gender preferences, gendered occupational segregation and economic disparities independent of partnerships and parenthood. Further qualitative and quantitative research is needed to disentangle these influences. For the latter, the pairfam study offers the opportunity to examine attitudes, satisfaction and preferences concerning time use and employment situation as well as partnership and family. Additionally, individual-level panel models would be desirable to confirm and extend the results presented. These panel models could control for unobserved heterogeneity and associated endogenous selection effects, such as career orientation and preferences regarding place of residence and high mobility. It would also be conceivable to differentiate between LDC, shuttles and vari-mobiles, as these differ from one another despite both being a way to overcome difficulties in reconciling professional and private life. Moreover, it would be interesting to compare the correlations identified here for Germany with other countries, e.g., Central and Eastern European countries, where the gender pay gap is less pronounced and the share of women in full-time work is higher (European Institute for Gender Equality 2014; Boll and Lagemann 2018).

## Data Availability

This paper uses data from the German Family Panel pairfam, release 10.0.0, which is accessible to the scientific community as scientific use file for scholarly analyses (https://doi.org/10.4232/pairfam.5678.10.0.0).

## Footnotes

1 Sometimes also called extreme commuting (Maoh and Tang 2012; Ravalet et al. 2015).

2 Although the term "long-duration commuter" would be more appropriate, we use the term “long-distance commuter” for reasons of consistency.

3 None of the survey waves provides information whether the partner's workplace changes or not. The same applies to the frequency and duration of commuting from the second residence. In contrast, the other data (nights spent away from home for work-related reasons, the frequency and duration of commuting from the main residence, and the number of nights spent at the temporary residence) were surveyed at irregular intervals and partly in different waves.

4 0.1% of anchors with children live in a same-sex partnership. The proportion of anchors without children who live in a same-sex partnership is substantially higher (3.3%). Analyses that exclude these anchors do not lead to noteworthy changes in the results. For this reason, the analyses include also anchor in same sex-partnerships.

5 Question posed: “On average, how long does this commute take you?”.

6 In 75% of cases, the anchor’s perception corresponds to the partner's assessment. The deviations are mainly between “egalitarian” and “mainly anchor” or “mainly partner”. Only 1% of the couples estimate their share of the division of labor to be exactly the opposite (“mainly anchor” vs. “mainly partner”).

7 Excluding "division of childcare" in Model 1, the Exp(B) of gender is 0.215, and thus at about the same level as in Model 2.

8 Regressions without these variables, not reported here for the sake of brevity, confirm a positive significant correlation between a high vocational degree and high mobility.
